# Genome-Scale Metabolic Modeling Predicts Per- and Polyfluoroalkyl Substance-Mediated Early Perturbations in Liver Metabolism

**DOI:** 10.3390/toxics13080684

**Published:** 2025-08-17

**Authors:** Archana Hari, Michele R. Balik-Meisner, Deepak Mav, Dhiral P. Phadke, Elizabeth H. Scholl, Ruchir R. Shah, Warren Casey, Scott S. Auerbach, Anders Wallqvist, Venkat R. Pannala

**Affiliations:** 1 Department of Defense Biotechnology High Performance Computing Software Applications Institute, Defense Health Agency Research & Development, Medical Research and Development Command, Fort Detrick, MD 21702, USA; 2 The Henry M. Jackson Foundation for the Advancement of Military Medicine, Inc., Bethesda, MD 20817, USA; 3 Sciome LLC, Research Triangle Park, NC 27709, USA; 4 Division of Translational Toxicology, National Institute of Environmental Health Sciences, Research Triangle Park, NC 27709, USA

**Keywords:** PFAS, liver metabolism, genome-scale metabolic modeling, BMD analysis

## Abstract

Per- and polyfluoroalkyl substances (PFASs) are widespread in the environment, bioaccumulate in humans, and lead to disease and organ injury, such as liver steatosis. However, we lack a clear understanding of how these chemicals cause organ-level toxicity. Here, we aimed to analyze PFAS-induced metabolic perturbations in male and female rat livers by combining a genome-scale metabolic model (GEM) and toxicogenomics. The combined approach overcomes the limitations of the individual methods by taking into account the interaction between multiple genes for metabolic reactions and using gene expression to constrain the predicted mechanistic possibilities. We obtained transcriptomic data from an acute exposure study, where male and female rats received a daily PFAS dose for five consecutive days, followed by liver transcriptome measurement. We integrated the transcriptome expression data with a rat GEM to computationally predict the metabolic activity in each rat’s liver, compare it between the control and PFAS-exposed rats, and predict the benchmark dose (BMD) at which each chemical induced metabolic changes. Overall, our results suggest that PFAS-induced metabolic changes occurred primarily within the lipid and amino acid pathways and were similar between the sexes but varied in the extent of change per dose based on sex and PFAS type. Specifically, we identified that PFASs affect fatty acid-related pathways (biosynthesis, oxidation, and sphingolipid metabolism), energy metabolism, protein metabolism, and inflammatory and inositol metabolite pools, which have been associated with fatty liver and/or insulin resistance. Based on these results, we hypothesize that PFAS exposure induces changes in liver metabolism and makes the organ sensitive to metabolic diseases in both sexes. Furthermore, we conclude that male rats are more sensitive to PFAS-induced metabolic aberrations in the liver than female rats. This combined approach using GEM-based predictions and BMD analysis can help develop mechanistic hypotheses regarding how toxicant exposure leads to metabolic disruptions and how these effects may differ between the sexes, thereby assisting in the metabolic risk assessment of toxicants.

## 1. Introduction

Per- and polyfluoroalkyl substances (PFASs) are human-made chemicals containing one or more perfluoroalkyl moieties (C*_n_*F_2*n*+1_). The perfluoroalkyl moieties enhance the hydrophobic and oleophobic properties of the chemicals and make them useful in consumer products, such as food packaging, cosmetics, cookware, and aqueous film-forming foams [1,2,3,4,5]. Ingestion of PFAS-contaminated food and water is the primary source of exposure in humans, followed by inhalation. Although dermal absorption is possible, its occurrence is considered low compared to oral and inhalation-based exposures, although more studies are needed [1]. The functional “head” group and the C-F chain length characterize PFAS chemicals [4,6]. The most commonly found PFASs contain a carboxylic acid (perfluoroalkyl carboxylic acids, PFCAs) or a sulfonic acid (perfluoroalkyl sulfonates, PFSAs) functional group. Long-chain PFASs include PFCAs with at least eight carbons and PFSAs with six or more carbons [6]. PFOA (perfluorooctanoic acid) and PFOS (perfluorooctanesulfonic acid), two of the most well-studied long-chain PFASs, contain a carboxylic acid and a sulfonic acid functional group, respectively. PFASs have gained interest as chemicals of concern due to their widespread use, chemical stability, bioaccumulation, and association with multiple adverse human health outcomes, including thyroid dysfunction, increased risk of cancer, neurotoxicity, and diminished reproductive health [1,5,7,8,9,10,11,12]. Chain length and functional group attachments are known to influence the bioaccumulation and effects of PFAS chemicals [3,13,14]. Furthermore, several precursors of PFASs, such as fluorotelomers, are gaining interest due to their ability to bio-transform into perfluoroalkyl acids, leading to their increased bioaccumulation in various organisms and in the environment [4,15,16,17]. Although some adverse effects of PFASs have been linked to peroxisome proliferator-activated receptor and constitutive androstane receptor activation [3], other effects, in particular those on the adaptive immune system, are poorly characterized at a mechanistic level [18].

PFASs are known to accumulate in the liver and have been associated with liver damage and disease, including non-alcoholic fatty liver disease (NAFLD), steatosis, and hepatocellular carcinoma [3,4,11,17,19,20,21,22,23,24,25,26,27,28]. Various studies have also reported sex-based PFAS outcomes [11,21,27,29,30,31,32], making it important to investigate PFAS-induced effects in the liver and in both sexes. Omics-based methodologies, such as transcriptomics and metabolomics, are routinely used to probe the perturbations in liver metabolism to understand the mechanisms of PFAS toxicity. For example, metabolic methods, such as serum metabolomics, can measure metabolite levels and help in assessing the progression of liver injury [33,34,35]. However, metabolomic-based analyses often face the challenges of reproducibility and a lack of well-defined annotations for metabolite identification. Furthermore, it is very difficult to link metabolomic measurements with the mechanisms of toxicity [36]. In contrast, transcriptomic methods can measure changes in gene expression and can be linked to known molecular pathways using various pathway-enrichment analysis platforms. However, these pathway-enrichment methods do not consider the interaction of multiple genes that take part in catalyzing metabolic reactions and the connectivity between the reactions for a quantitative understanding of the mechanisms behind the perturbations [37,38,39].

Genome-scale metabolic modeling is a systems-level approach that captures cellular dynamics by integrating gene and metabolic information into genome-scale metabolic models (GEMs), which are mathematical representations of all the metabolic bio-transformations within a cell or organism. The models are comprised of metabolites and reactions in the form of a stoichiometric matrix, which considers the connectivity between the reactions. The models also contain gene–protein–reaction (GPR) rules that connect genes to reactions and allow for integration of omics measurements to simulate cellular phenotypes using constraint-based algorithms, such as flux balance analyses [40]. In addition to containing metabolism-associated data, GEMs group reactions into metabolic subsystems (such as central carbon metabolism, electron transport chain, and fatty acid oxidation) and assign reactions to their cellular compartments (cytoplasm, nucleus, mitochondria, etc.). GEMs, along with constraint-based algorithms, find applications in research that require the study of cellular metabolism, such as for developing novel strains to produce value-added chemicals, generating hypotheses, identifying novel drug targets, and understanding diseases [41,42,43,44,45]. Recently, GEMs have helped us understand toxicity responses in various cellular systems [46,47,48,49,50,51,52], but most of these studies focused on predicting changes in metabolite secretions or studying differences in selected liver metabolic tasks and subsystems, based on the differentially expressed genes, but did not include dose–response studies. Furthermore, the approach of Moore et al. [52] relied on using metabolic models that meet the requirement of at least 40% original maximum biomass flux, suggesting the need for a well-defined biomass function that may not always be available and could vary by experiment.

One of the primary objectives of chemical risk assessment is to identify the critical dose, called the benchmark dose (BMD), beyond which exposure is toxic. The BMD modeling approach fits mathematical models to dose–response data to identify the dose at which the response is associated with a benchmark response (such as a 10% increase from the controls) [53]. The U.S. Environmental Protection Agency (EPA) released a benchmark dose software (BMDS) that aids in the estimation of BMDs using dose–response data as input [54]. The methods embodied in the BMDS have been adapted to the evaluation of gene expression, allowing for estimates of biological function potency [55,56]. Many studies have applied the BMD modeling method; however, none have used it to identify reference doses for individual metabolic pathway alterations while considering the interconnectedness of the metabolic network. GEM-based modeling allows us to predict metabolic reaction flux distributions based on gene expression data, which can be used to identify BMDs for metabolic pathways in the liver.

To overcome the limitations of individually performing transcriptomics-based or GEM-based analyses, an approach that combines both was applied. By integrating the two methods, the transcriptomic data function as the additional constraints that GEMs require for predicting accurate metabolic flux states. There are several algorithms available to integrate transcriptomics with GEMs, and we direct the reader to a review by Sen et al. [57] for more details on these algorithms. However, most of these algorithms predict the activation of reactions rather than their flux rate. The linear programming-based algorithms that predict flux values (such as flux balance analysis) use an objective function to constrain the solution space and could have multiple alternate optimal solutions due to the presence of loops within the network, making it harder to identify a mechanism that represents the biological phenotype. However, the Pheflux algorithm, in contrast to other constraint-based algorithms, uses the principles of maximum entropy and minimized sum of fluxes to predict a set of genome-scale reaction rates (i.e., the fluxome) that closely match the gene expression pattern based on the GPR rules defined in the metabolic model [58]. Pheflux is designed to predict the fluxome solution that is the most likely to be biologically observed, and, importantly, the algorithm does not require an objective function, making it advantageous in our analysis because the objective of cellular adaptations to toxic exposures is not yet completely known. We hypothesize that the PFAS chemicals studied here will induce sex- and dose-dependent metabolic alterations that connect the exposures to liver injury phenotypes, such as steatosis, and that our combined GEM- and transcriptomic-based approach will capture these alterations.

In this study, we utilized the transcriptomic measurements from a National Institute of Environmental Health Sciences (NIEHS) 5-day exposure study [59,60,61], where Auerbach et al. exposed male and female rats to a daily dose of a PFAS chemical for 5 days, and then extracted liver tissue samples and measured gene expression using S1500+ TempO-Seq [62]. Following our hypothesis, the gene expression measurements were integrated with an updated rat GEM to predict individual rat liver phenotypes using the algorithm Pheflux [58]. We analyzed the Pheflux-predicted fluxomes to understand how male and female rats differed at the control level and how each sex responded to the PFAS chemicals, and compared fluxomes across different conditions to understand how exposure dose and sex can alter metabolism in the liver. Finally, the fluxome predictions were used to estimate BMDs for each subsystem alteration in response to each PFAS chemical in each sex. Our analysis revealed the key metabolic pathway alterations induced by PFAS exposure in male and female rat livers, including subsystems from lipid, amino acid, and energy metabolism. Our BMD analysis further revealed that males were more sensitive to the PFAS chemicals and that 6:1 fluorotelomer alcohol (FTOH) induced the earliest changes. We propose this metabolic modeling and BMD approach as a novel metabolic risk assessment tool that describes the transcriptome-driven metabolic changes induced by chemical exposures.

## 2. Materials and Methods

### 2.1. Animal Exposure Experiments and Transcriptomics

To measure the transcriptomic responses to PFAS chemicals, male and female Sprague Dawley rats were exposed to various doses of three PFAS compounds [6:1 FTOH, 10:2 FTOH, and perfluorohexanesulfonamide (PFHxSAm)] [59,60,61]. Two of the PFASs, 10:2 FTOH and PFHxSAm, were sourced from SynQuest Laboratories, Inc. (Alachua, FL, USA), and 6:1 FTOH was obtained from Apollo Scientific, Ltd. (Stockport, UK). At the time of the experiments, these PFAS chemicals were classified as data-sparse, with no in vivo toxicological information. Auerbach et al. used median lethal dose (LD_50_) predictions from the OPEn structure–activity/property Relationship App (OPERA) [63] as well as point-of-departure predictions from the U.S. EPA to select the test doses for each chemical. Table 1 provides a brief summary of the selected chemicals and the dosages used in the experiments.

**Figure 1:**
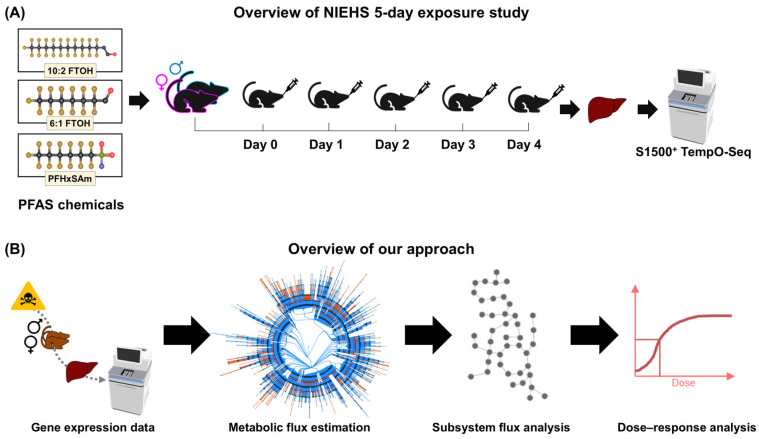
Overview of the workflow of this study. (**A**) The National Institute of Environmental Health Sciences (NIEHS) 5-day rat exposure study. (**B**) Our computational approach for metabolic risk assessment, including integration of transcriptomics with a rat genome-scale metabolic model (network visualization from [66]) for prediction of the fluxome using Pheflux [58], processing fluxome predictions to predict metabolic subsystem alterations in PFAS-exposed rats compared to the control rats, and dose–response analysis to calculate benchmark doses of each chemical for each metabolic subsystem alteration. FTOH, fluorotelomer alcohol; PFASs, per- and polyfluoroalkyl substances; and PFHxSAm, perfluorohexanesulfonamide.

Rats were randomly assigned to the control or dose groups, where each dose group contained 5 rats of each sex, and the control group contained 10 rats of each sex. The rats received the assigned dose of chemical or vehicle via oral gavage for 5 consecutive days starting on Day 0. On Day 5 (24 h after the final dose administration), the rats were euthanized, and liver tissue samples were collected for RNA sequencing. Total RNA was extracted from the liver samples and sequenced using the S1500+ TempO-Seq platform [62,64]. The S1500+ TempO-Seq read alignment, normalization, log_2_ transformation, and extrapolation to the whole transcriptome (~17k genes) were performed using the GeniE software (version 3.0.4) [65] as described in Hari et al. [32]. Figure 1A summarizes the NIEHS experiments. For a complete description of the experimental studies, we direct the reader to the published NIEHS reports [59,60,61]. We used the extrapolated, whole transcriptome dataset for the rest of this study.

### 2.2. Computational Approach for Metabolic Risk Assessment of PFAS Chemicals

To estimate the metabolic risk of each PFAS chemical, we developed and applied a computational approach that combines GEM and BMD modeling. Figure 1B depicts the computational approach used in this study. Briefly, the Pheflux algorithm [58] was first applied to integrate the gene expression data with a rat GEM, and the predicted fluxes were used to estimate metabolic subsystem alterations induced by each dose of each PFAS used in the rat experiments. Then, the subsystem alterations between the control and PFAS-treated rats were compared to assess the effects of exposure, and the alterations between male and female PFAS-exposed rats were compared to identify sex-dependent effects. Finally, we used the subsystem alterations to predict the BMD at which each of the chemicals alters fluxes in the subsystem. We describe each step of the workflow in the following sections.

### 2.3. Rat Genome-Scale Metabolic Model

**Figure 2:**
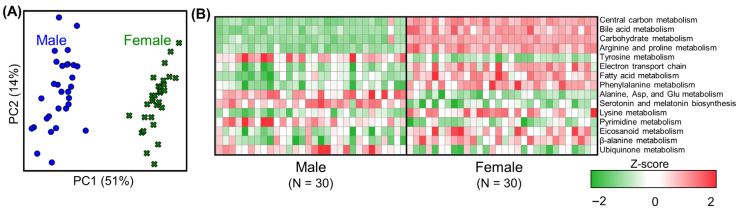
Metabolic network analysis of sexual dimorphism in the control rat livers. (**A**) Principal component (PC) analysis of male (blue circles) and female (green crosses) control rats. (**B**) Heatmap showing z-scores for the mean metabolic fluxes of the top 15 sex-dependent subsystems in each rat with respect to the mean subsystem fluxes across all the control rats.

Prediction of metabolic pathway alterations using transcriptomics requires a network that contains all the metabolic reactions associated with the genes that produce the proteins catalyzing the reactions. To predict the metabolic alterations resulting from PFAS exposure, we used the latest version of the rat metabolic network model, i.e., iRnov4.2, which was derived from previous versions of iRno [46,48,67,68] and the latest RAT-GEM [69]. The previous version of iRno (iRnov4.1) [46] contained 13,043 reactions, 8414 metabolites, and 3102 genes. For this study, we used the same network configuration as iRnov4.1 and modified only the subsystem organization of the reactions. In iRnov4.1, each reaction belonged to one of 76 subsystems. We reconciled the reaction subsystem associations using annotations from the recently updated RAT-GEM [69]. Information from the KEGG pathway database [70,71] was used to further classify subsystems into their major pathways, such as amino acid metabolism, lipid metabolism, and vitamin and cofactor metabolism. This updated version of the model (iRnov4.2) thus contains reactions organized into 58 metabolic subsystems and 27 major pathways. Figure 2B contains a global network visualization of iRnov4.2 in the metabolic flux estimation step that was generated using Fluxer [66] (https://fluxer.umbc.edu, accessed on 31 July 2025). Supplementary File S1 details the subsystem mappings for *iRnov4.2*, and Supplementary File S2 contains the metabolic model in .xml format. We used the Python package *cobrapy* (0.29.1) [72] to read the metabolic network model in order to calculate the metabolic subsystem alterations.

### 2.4. Fluxome Prediction Using the Pheflux Algorithm

The Pheflux algorithm developed by González-Arrué et al. [58] was used to predict the fluxome from the metabolic network and transcriptome. Briefly, the algorithm uses the principle of maximum entropy to estimate the flux on a reaction (*v*). For any metabolic state represented by a transcriptome measurement, the algorithm assumes a steady-state condition (Equation (1)):

(1) Sv = 0

(2) LB ≤ v ≤ UB

where S is the stoichiometric matrix of a metabolic network containing N reactions and M metabolites. Each reaction was constrained by using a lower bound (LB) and an upper bound (UB), as defined in the original iRnov4.1 model. The constraints on exchange reactions, which represent the movement of metabolites from the extracellular to the intracellular space, were set to a default value of 1000 mmol/g dry cell weight/h in both directions:

(3) -1000 ≤ v ≤ 1000

The polytope of all possible fluxomes (℘) is described as a set of all possible reaction fluxes satisfying Equations (1) and (2):

(4) $$℘\;=\;\left\{v\;\epsilon \;R^{N}|\;Sv\;=\;0,\;LB\text{ ≤ }v\text{ ≤ }UB\right\}$$

where $R^{N}$ is a vector of all reaction fluxes.

Pheflux estimates the fluxome by solving the optimization problem to minimize the Kullback–Leibler distance between the fluxome and transcriptome, ensuring that the distribution of predicted reaction fluxes is closest to the measured transcriptome while applying metabolic model constraints. Thus, the algorithm applies the constraints in Equations (1) and (2) and solves the following objective function:

(5) $$min\text{:}D_{KL}(P\text{∥}Q)\;=\;\sum_{i=1}^{N}P(v_{i})log\frac{v_{i}}{g_{i}}$$

where *D_KL_* represents the Kullback–Leibler distance, *P* denotes the probability distribution of the fluxome, *Q* represents the probability distribution of gene expression per reaction, and *g* and *v* denote the expression of genes and flux on the *i*th reaction, respectively. Detailed derivations and a full explanation of the Pheflux algorithm are given in the original publication [58].

To calculate the metabolic subsystem flux in each rat (*μ_subsystem_*), the absolute reaction fluxes of all reactions in the subsystem were averaged over the total number of reactions in that subsystem (*r*):

(6) $$\mu_{subsystem}\;=\;\frac{\sum_{i=1}^{N}{|v}_{i}|}{r}$$

The response to a dose was estimated using an average of the individual rat subsystem fluxes to estimate, as shown below:

(7) $$\mu_{subsystem,dose}\;=\;\frac{\sum_{i=1}^{n}v_{subsystem,i}}{n}$$

where n represents the number of rats in the dose group and $v_{subsystem,i}$ denotes the subsystem flux in the ith rat.

A z-score was used to calculate the change in flux, with respect to the controls, per subsystem:

(8) $$z\;=\;\frac{\mu_{subsystem,dose}\;-\;\mu_{subsystem,control}}{\sigma_{subsystem,control}}$$

Because the S1500^+^ extrapolated transcriptome dataset contained gene expression data that were log_2_-normalized, we performed the inverse of the log_2_ function and provided the raw gene counts as input to Pheflux. Furthermore, because the gene expression dataset already contained genes in the ENTREZ ID namespace, which matches the gene IDs in the metabolic model, we did not perform any gene ID conversion when using the expression dataset.

### 2.5. Principal Component Analysis of Subsystem Fluxes

We applied a principal component analysis (PCA) to reduce the number of dimensions in the fluxome dataset into two principal components, with each component containing the ranking of each feature (subsystem flux) based on its contribution to the variance between the datasets. Here, the PCA was performed, using the *PCA* function from the *sklearn* Python library, to identify the key metabolic subsystems that separate the rats in each chemical dose group. Each input data point represented an individual rat with metabolic subsystem fluxes (calculated from Equation (5)) as features. Thus, each chemical- and sex-based PCA included 55 data points (10 controls, 5 rats per exposure group) and 45 features (metabolic subsystems). We also performed a sex-aggregated PCA to identify the contribution of features selected from the chemical- and sex-based analyses. In the sex-aggregated PCA, there were 325 data points (30 controls of each sex, 135 PFAS-exposed female rats, and 130 PFAS-exposed male rats).

### 2.6. Computational Resources

The scripts for the Pheflux algorithm (v1.0.1) were downloaded from GitHub (https://github.com/mrivas/pheflux, accessed on 31 July 2025) and run on Python (3.12.7). The Pheflux results were saved as .csv tables. The *pandas* (2.2.2) Python library was used to read and process the Pheflux-generated reaction flux tables, the *Seaborn* (0.13.2) library was used to plot the heatmaps, and the Python plotting libraries *matplotlib* (3.9.2) and *plotly* (5.24.1) were used for the PCA plots.

### 2.7. Benchmark Dose Analysis

We used the Python package *pybmds* (24.1) [73] to estimate the BMDs for each pair of chemical and metabolic subsystem responses, and we further performed the analysis independently for each sex. We provided individual rat subsystem fluxes for each dose as continuous data for BMD prediction and fit the data to five types of dose–response models: linear, polynomial, power, hill, and exponential. We used the default benchmark response (BMR) of one standard deviation (SD), relative to the control, to predict the BMDs and kept all the default dose–response model parameter values.

The *pybmds* package follows the recommended logic described by Wignall et al. for BMD modeling [53]. Thus, the program classifies each model as unusable, questionable, or viable. We consecutively deleted the highest dose when the existing set of doses and responses produced a non-viable BMD model (questionable and unusable models), until the BMD modeling function either returned a viable model or there were only three doses left to input to the function. Here, we report the latter case, i.e., modeling with three dose–response groups, as “no viable model was found.”

## 3. Results

### 3.1. Metabolic Flux Analysis to Quantify Liver Metabolic Activity Using Gene Expression Data

We applied the Pheflux algorithm to predict the metabolic flux of each reaction in iRnov4.2 using gene expression data from each untreated and chemical-treated rat as input. Each Pheflux optimization returned the metabolic flux for the 13,043 reactions that are divided into 58 subsystems in iRnov4.2. To calculate the flux-based activity in each subsystem for each rat, the absolute fluxes of all the reactions in the subsystem were averaged using Equation (5) and then that value was normalized by the sum of all the absolute fluxes in the rat, resulting in a table with 58 rows and 301 columns that represent the subsystems and rats, respectively. We removed all the subsystems (miscellaneous, artificial reactions, pool reactions, biomass, exchange, isolated, and transport) that contained reactions associated with less than two genes as well as the subsystems with fluxes that did not change across the rats (SD = 0), resulting in a total of 45 metabolic subsystems that were associated with at least two genes and showed a change in flux across the rats. Subsequently, the generated individual reaction fluxes at the subsystem level from each rat were used to quantify the liver metabolic activity across chemicals, sex, and conditions using the PCA and BMD analyses.

### 3.2. Metabolic Analysis of Liver Metabolism in Untreated Rats (Controls)

The metabolic flux predictions for the male and female control rats were used to assess whether our network modeling approach captured sexual dimorphism at the metabolic level. The metabolic activity at the subsystem level was estimated using the GEM-predicted reaction fluxes from each rat in the male and female groups. Figure 2A shows the PCA analysis using the reaction flux distributions at the subsystem level, which clearly indicates a sex-dependent segregation of metabolic activity between the male (circles) and female (crosses) groups as well as differences in liver metabolism between the sexes. To discover the potential contributing factors behind the observed differences, we extracted the top 15 metabolic subsystems that significantly contributed to the largest variation observed along principal component 1 (PC1, with 51% variance). Figure 2B shows a heatmap of the liver metabolic activity for the top 15 metabolic subsystems identified based on their weighted contribution in the PCA analysis. Here, we used a z-score-based calculation to compare the reaction fluxes at the subsystem level between the two sexes, with positive (red) and negative (green) values indicating reaction fluxes that were higher and lower than the average subsystem flux across all the control rats, respectively. The top subsystems contributing significantly to the observed metabolic differences between the male and female control rats included central carbon metabolism, bile acid metabolism, carbohydrate metabolism, arginine and proline metabolism, and tyrosine metabolism. Furthermore, the positive z-score values in the central carbon metabolism, electron transport chain, and carbohydrate metabolism subsystems suggested that female rats have higher metabolic activity in these pathways compared to male rats. Similarly, the results showed higher metabolic activity in the bile acid metabolism, fatty acid metabolism, and eicosanoid metabolism subsystems in female compared to male rats. In contrast, the metabolic activity in amino acid metabolism (such as tyrosine, alanine, aspartate, and glutamate) and serotonin and melatonin metabolism subsystems was higher in male compared to female rats. These differences suggest that male and female rats have different liver-based metabolic needs at the control level and that our network modeling approach was able to capture these differences.

### 3.3. Effect of PFAS Exposure on Sexual Dimorphism in Male and Female Rat Livers

To determine how PFAS exposure affects sex-dependent metabolism in rats, a combined PCA analysis for each chemical was performed by providing the reaction fluxes at the subsystem level for each control and chemical-exposed group (male and female) together as input features. Our results revealed that the rats exposed to PFASs primarily clustered by sex and, within each sex-based cluster, by exposure dose (Supplementary Figure S1). The separation between dose-based clusters differed for males and females, suggesting differences in dose sensitivity between the sexes. Overall, these results suggest that rats maintain sexual dimorphism even after exposure to various PFAS concentrations.

### 3.4. Analysis of PFAS Dose-Dependent Alterations in Rat Liver Metabolism

**Figure 3:**
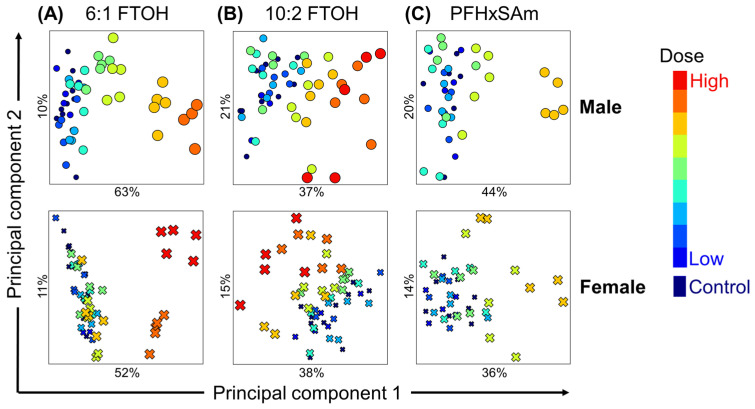
Identification of PFAS-induced metabolic pathway perturbations in male (top) and female (bottom) rats using principal component analysis. (**A**) Exposure to 6:1 fluorotelomer alcohol (FTOH). (**B**) Exposure to 10:2 FTOH. (**C**) Exposure to perfluorohexanesulfonamide (PFHxSAm). Circles denote male rats, and crosses denote female rats. Color gradient corresponds to exposure dose.

To understand the overall effect of PFAS exposure on liver metabolism and identify the metabolic subsystems altered for each sex and each PFAS chemical, we calculated the mean subsystem reaction fluxes for each rat in the control and PFAS-exposed groups and performed a PCA analysis. Figure 3 shows an overview of the dose-dependent alterations in liver metabolic activity for each PFAS chemical in male (top panel) and female rats (bottom panel). The PCA plots show that PFAS exposure dose-dependently altered liver metabolism, with a clear separation of low-dose (blue) and high-dose (red) PFAS exposures. The results also show that high doses of 6:1 FTOH induced the largest change for male (63%) and female rats (52%) from their respective controls (Figure 3A), compared to 10:2 FTOH (Figure 3B; male 37% and female 38%) and PFHxSAm (Figure 3C; male 44% and female 36%). Furthermore, the PCA plots revealed that male rats generally showed a larger dose-dependent variance in metabolic perturbations than females.

### 3.5. Metabolic Pathways Affected by PFAS Chemicals

**Figure 4:**
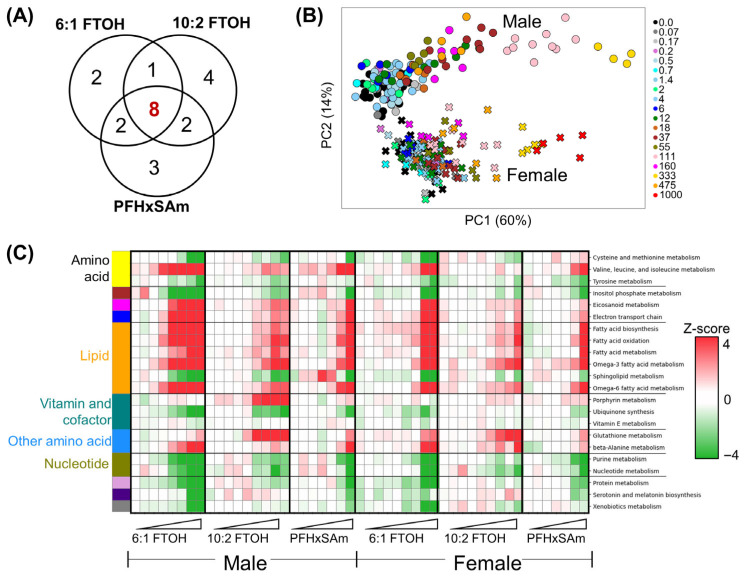
The most-altered metabolic subsystems across different PFAS exposures. (**A**) Venn diagram of the most-altered subsystems for each PFAS. (**B**) Principal component (PC) analysis of rat fluxes in the top 22 PFAS-associated pathways. (**C**) Z-scores for average metabolic subsystem fluxes per dose group with respect to the controls. Triangles depict increasing PFAS doses. The colored ribbon on the left of the heatmap denotes the super-pathway classifications of the subsystems: yellow—amino acid metabolism; brown—inositol phosphate metabolism; pink—eicosanoid metabolism; blue—electron transport chain; orange—lipid metabolism; sage green—vitamin and cofactor metabolism; sky blue—metabolism of other amino acids; olive—nucleotide metabolism; lavender—protein metabolism; purple—serotonin and melatonin metabolism; gray—xenobiotic metabolism. FTOH: fluorotelomer alcohol; PFHxSAm: perfluorohexanesulfonamide.

To understand the dose-dependent metabolic changes induced by PFAS exposure, we extracted the top 10 most-altered metabolic subsystems based on the features separating the low-dose and high-dose exposures in the PCA analyses shown in Figure 3. A complete list of the most-altered subsystems and their ranking as detected along PC1 for each chemical is provided in Supplementary Table S1. Of these, fatty acid oxidation, electron transport chain, and fatty acid biosynthesis were common for all three chemicals and both sexes, suggesting that alterations in these three subsystems are central to the PFAS-induced adverse effects in both sexes. To identify the common subsystem alterations across the PFAS types, we created a set of the most-altered subsystems for each chemical that combines the male and female most-altered subsystems for that chemical. Figure 4A shows a Venn diagram comparing the sets of most-altered subsystems for each chemical and the subsystems common between them. The combination of all three sets included 22 metabolic subsystems, with eight subsystems common to all three PFAS chemicals. Subsequently, a PCA using the mean fluxes in only these 22 subsystems for both male and female rats was used to assess their dose-dependent variation. Figure 4B shows the PCA results, with colors indicating exposure doses, circles representing male rats, and crosses representing female rats, which revealed that alterations in these 22 subsystems account for a large proportion of the difference between rats in the low-dose and high-dose exposure groups. Furthermore, the PCA plot showed sex-dependent responses in the 22 subsystems, with dose-based separation more pronounced in the male rats, suggesting that the dose sensitivity to these chemicals is also sex dependent.

To investigate whether PFAS exposure increased or decreased the flux-based activity in each of these metabolic subsystems, the change in mean subsystem fluxes with respect to the controls as a z-score was calculated and estimated using Equation (7). The z-score represents the number of SDs by which the mean subsystem flux in a chemical-exposed group changes compared to the corresponding control group. Positive and negative z-scores for a pathway imply that the fluxes in the chemical-exposed group were higher and lower than in the control rats, respectively. Figure 4C shows a heatmap of the z-scores for the 22 most-altered subsystems, with positive (red) and negative (green) z-scores indicating increased and decreased metabolic activity, respectively. The metabolic subsystems were grouped based on their super-pathway classifications (i.e., metabolism of amino acids, lipids, vitamins and cofactors, nucleotides, and other amino acids). The heatmap revealed that the alteration direction for each subsystem was similar between the chemicals and between the sexes. Furthermore, most of the alterations were dose-dependent, and males showed alterations earlier than females. Exposure to high doses of PFASs decreased the activity in the amino acid metabolism subsystem, except for metabolism of the branched-chain amino acids (valine, leucine, and isoleucine), which showed a dose-dependent increase in activity. However, the mean fluxes in metabolism of cysteine and methionine increased in females in response to high doses of PFHxSAm, which was different from the trend observed for the other chemicals and in male rats. The lipid metabolism subsystems mostly showed an increase in activity for both male and female rats compared to the controls, except for sphingolipid metabolism, which showed decreased fluxes at high-dose exposures. For the vitamin and cofactor subsystem, porphyrin metabolism showed an increased activity only in response to 10:2 FTOH and PFHxSAm, ubiquinone synthesis showed a dose-dependent decrease in flux-based activity at high doses of all three chemicals in male rats, while vitamin E metabolism decreased consistently compared to the controls only in males exposed to 6:1 FTOH. Exposure to the PFAS chemicals caused a decrease in activity in the nucleotide metabolism subsystems, whereas the metabolism of other amino acids (glutathione and beta-alanine) showed an increase in activity. For the other 22 most-altered subsystems, apart from eicosanoid metabolism and the electron transport chain, the mean flux decreased compared to the controls.

To identify the metabolic perturbations that were significantly altered after all the PFAS exposures, we performed a Mann–Whitney U test to compare the subsystem fluxes in the control and chemical-exposed rats. Supplementary Figure S2 shows a heatmap of the z-score changes for the eight common PFAS-altered subsystems. Our results revealed significant changes compared to the controls in response to high doses of 6:1 FTOH in both male and female rats (*p*-value < 0.05). For high-dose 10:2 FTOH and PFHxSAm, although some of the subsystem changes were not significant, their average direction of change was consistent, suggesting that the tests likely failed due to intra-sample variation within the dose group.

### 3.6. Correlation Between Male and Female Responses to PFAS Exposures

**Figure 5:**
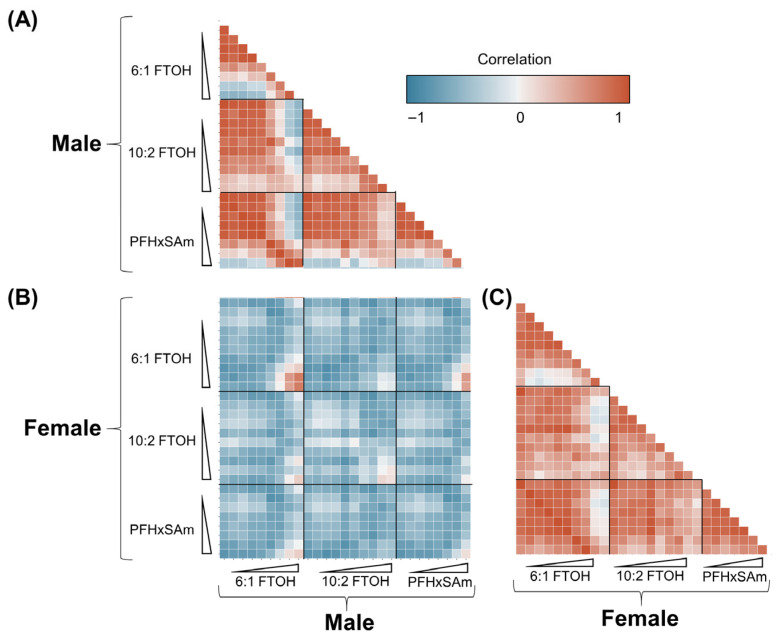
Correlation of metabolic fluxes between PFAS-exposed male and female rats. Blue and red boxes represent negative and positive correlations between groups, respectively. (**A**) Correlation between male rats. (**B**) Correlation between male and female rats. (**C**) Correlation between female rats. Triangles depict increasing PFAS doses. FTOH: fluorotelomer alcohol; PFHxSAm: perfluorohexanesulfonamide.

To assess the similarity in PFAS-induced metabolic alterations between males and females, we plotted a correlation map comparing the average fluxes in all the subsystems in each chemical dose group in male and female rats. Figure 5 shows the correlation plots, with negative correlations in blue and positive correlations in red. Figure 5B shows the correlation between male and female rat fluxomes at each dose of exposure, indicating that the metabolic fluxes were negatively correlated (different) between the sexes at low doses but positively correlated (similar) for high-dose exposures. Furthermore, when exposed to the same chemical, the metabolic responses of male and female rats (Figure 5A,C, respectively) were correlated based on the exposure dose: low-dose exposures were positively correlated with each other and negatively correlated with the high-dose exposures, and vice versa. For each exposure in a single sex, the doses at which the correlations separated roughly indicate the metabolic alteration points of departure. Some of the dose responses across chemicals were also positively correlated at high doses, which suggests that these chemicals induced similar responses for high-dose exposures.

### 3.7. Benchmark Doses of PFAS Common Metabolic Alterations

To identify the doses that represent the metabolic alteration points of departure corresponding to a BMR (>1 SD) from the control rats, we performed sex-based BMD modeling to determine the mean fluxes in each subsystem in response to each chemical. The default BMD modeling workflow was applied, as described in Section 2. With the default settings, the BMD modeling software found 208 viable models (listed in Supplementary File S3). The results included the predicted BMDs for each subsystem in response to each chemical and a confidence interval (BMDL–BMDU) for the BMD, where BMDL represents the lower confidence limit and BMDU the upper limit. The list of BMD results was filtered to remove any models that predicted BMD or BMDU values outside the doses that were input to the BMD function, as these represent results fitting the model curves rather than the real exposure data. This filtering resulted in 130 viable models: 59.2% exponential, 14.6% polynomial, 11.5% Hill, 7% linear, and 7.7% power models.

Table 2 summarizes the BMDs predicted for each chemical and sex for the 22 PFAS-associated subsystems identified from the PCA analysis. Of the 22 subsystems, fatty acid oxidation and purine metabolism had viable models for all chemicals and both sexes, but fatty acid oxidation was the only PFAS common pathway. Furthermore, of all the sex and chemical-exposure combinations, males exposed to 6:1 FTOH had the lowest BMDs for all the subsystems, suggesting that males exposed to this PFAS showed alterations earlier than when exposed to the other chemicals and even earlier than females exposed to any of the PFASs. The BMD of branched-chain amino acid metabolism (valine, leucine, and isoleucine) had the lowest BMD in response to 6:1 FTOH. The subsystem alterations in female rats exposed to 6:1 FTOH were higher than for males exposed to the same chemical, except for glutathione metabolism, which had a lower BMD for females. However, the BMD confidence interval (BMDL–BMDU) for this subsystem overlapped between the sexes, suggesting its perturbation to low doses of 6:1 FTOH is a sex-independent mechanism. The predicted BMDs for males and females exposed to 10:2 FTOH were similar for glutathione metabolism, purine metabolism, sphingolipid metabolism, and xenobiotic metabolism. The BMDL and BMDU values for these subsystems contained overlapping BMD ranges for males and females, suggesting that exposure to low doses of 10:2 FTOH induced similar responses in both sexes. Similarly, the BMDs predicted for rats exposed to PFHxSAm had overlapping ranges for cysteine and methionine metabolism, fatty acid oxidation, omega-6 fatty acid metabolism, purine metabolism, and ubiquinone synthesis in males and females.

**Figure 6:**
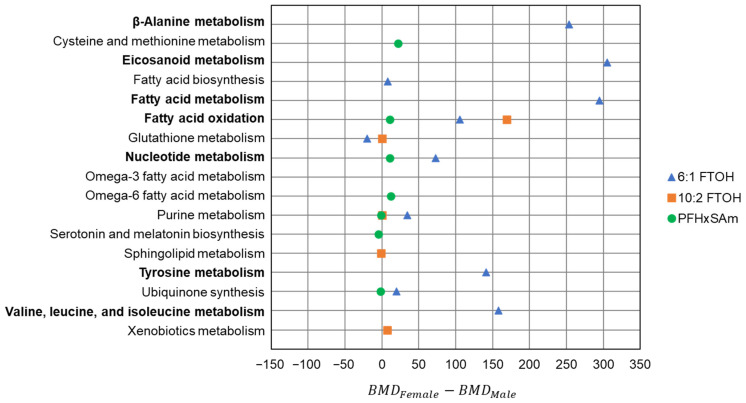
The difference between the male and female benchmark doses predicted for each chemical. Positive values indicate male sensitivity, and negative values indicate female sensitivity. Subsystem names in bold indicate the largest differences. FTOH: fluorotelomer alcohol; PFHxSAm: perfluorohexanesulfonamide.

To specifically compare the BMDs between the sexes, the difference pathways with BMD predictions for both males and females were visualized in a scatter plot. Figure 6 shows the differences in BMDs between female and male rats, with positive values indicating male sensitivity and negative values indicating female sensitivity. As shown in Figure 6, males were more sensitive to 6:1 FTOH exposure than females, particularly for beta-alanine metabolism, eicosanoid metabolism, fatty acid metabolism, fatty acid oxidation, nucleotide metabolism, tyrosine metabolism, and valine, leucine, and isoleucine metabolism. In contrast to 6:1 FTOH, both sexes showed a similar sensitivity to PFHxSAm and 10:2 FTOH, with most of their BMDs within 15 mg/kg of each other. Overall, these results indicate that the sexes show different sensitivities to different PFAS types.

## 4. Discussion

In this study, we developed a workflow that incorporates GEM and BMD modeling for metabolic risk assessment of chemicals using gene expression data. We applied the approach to analyze the metabolic risk of three PFAS chemicals in male and female rat livers and identified potential similarities and differences in the liver’s response to the PFAS chemicals. We integrated the gene expression data with a GEM to predict the flux-based activity in metabolic subsystems for each rat, compared the flux predictions between PFAS-exposed and unexposed rats to identify the subsystems altered due to PFAS exposure, and predicted the BMDs for each altered subsystem to determine their sensitivity.

The most-altered metabolic subsystems were identified by performing PCA for each chemical dose exposure in each sex and extracting the top features (subsystems) from each PCA. The PCA identified 22 PFAS-relevant pathways, with eight common to all three chemicals in this study (Figure 4). The results showed that most of the metabolic alterations were similar between the three PFAS chemicals, with most of the disrupted subsystems belonging to lipid, energy, and amino acid metabolism, and had the same direction of change (increase or decrease in flux activity with respect to the controls). However, comparing these changes across all the subsystems in the GEM revealed that PFAS exposure significantly decreased the metabolic activity in amino acid and nucleotide metabolism but increased the metabolic activity in lipid metabolism (Supplementary Figure S3). Specifically, with PFAS exposure, there was a consistent increase in metabolic activity in branched-chain amino acid metabolism (valine, leucine, and isoleucine) for both male and female rats compared to several other pathways in amino acid metabolism. We also found some differences in response based on the PFAS type. For example, exposure to 6:1 FTOH and PFHxSAm consistently increased the metabolic activity in tryptophan metabolism for both male and female rats, but decreased it with 10:2 FTOH. In contrast, there was an opposite behavior for lysine metabolism, with its activity increased in response to 10:2 FTOH but decreased or unchanged for the other two chemicals. Our analysis also identified protein and β-alanine metabolism alterations in response to individual PFASs, which likely resulted from changes in the individual amino acid subsystems. Several literature studies link these amino acid aberrations with PFAS exposure [11,74] as well as NAFLD and fibrosis [75,76,77]. Interestingly, Mardinoglu et al. used a GEM-based approach to compare hepatocyte activity between non-alcoholic steatohepatitis (NASH) and control patients and identified similar amino acid disruptions in the NASH patients, particularly the downregulation of serine [44,78].

Compared to changes in amino acid metabolism, there was a dose-dependent increase in the activity of most subsystems in lipid metabolism, with the activity of the majority of the subsystems consistently increasing as the dose increased. For example, for both male and female rats, several pathways in fatty acid metabolism, such as fatty acid oxidation, fatty acid biosynthesis, and omega-3 and omega-6 fatty acid metabolism, showed consistently increased metabolic activity with increasing PFAS doses, indicating potentially common mechanisms between males and females for PFAS exposure. However, there also were some chemical- and sex-specific changes in lipid metabolism, such as changes in the metabolism of bile acids, arachidonic acid, glycerolipids, sphingolipids, and steroids, in response to PFAS exposure (Supplementary Figure S3). Several other studies have also reported a decrease in bile acid metabolism due to PFAS exposure, particularly via the gene *Cyp7a1*, which codes for the enzyme (CYP7A1) that catalyzes the rate-limiting step of bile acid synthesis [3,21,29,30,79,80,81]. Our previous steatosis adverse outcome pathway-based analysis of the same dataset revealed that *Cyp7a1* decreased specifically in males [32]; however, the metabolic network analysis in this study predicted that females also showed a decrease in bile acid metabolism. It is possible that female rats reduce bile acid metabolism by a *Cyp7a1*-independent mechanism, but the current results and data are insufficient to validate this.

Cholesterol homeostasis in the liver includes conversion of cholesterol to bile acids via Cyp7a1. An impairment in this mechanism can thus lead to cholesterol accumulation and imbalance in the liver, contributing to steatosis and NAFLD [29,32]. The PFAS-induced disruption of bile acids has also been associated with hepatomegaly and cholestasis in mice [82,83]. Interestingly, our results showed an increase in bile acid metabolism in response to PFHxSAm in female rats, suggesting that the sulfonic acid attachment could be influencing bile acid metabolism in female rat livers. Furthermore, glutathione functions as an antioxidant and protects cells from oxidative stress and damage [84,85]. We thus hypothesize that the increase in glutathione metabolism predicted in our study is in response to the increase in fatty acid oxidation mechanisms. The glutathione responses to 10:2 FTOH were higher in both male and female rats compared to the other two PFASs, which could be attributed to 10:2 FTOH’s chain length.

Similar to our findings, other metabolomic studies have reported that metabolites of sphingolipid metabolism (such as ceramides and phosphosphingolipids) were altered in response to PFAS exposure. Ceramides, which are products of sphingolipid metabolism, take part in signaling and inflammation, contribute to the structural stability of cell membranes, and play a role in autophagy, cell proliferation, and immune responses [86]. Alterations in these metabolites could thus lead to increased inflammation and cellular damage [87], which are also reported consequences of PFAS exposure [88,89]. In addition, ceramides function in supporting mitochondrial homeostasis, and their dysregulation leads to oxidative stress and apoptosis [90]. Furthermore, ceramide imbalances have been associated with NASH development, liver fibrosis, and cirrhosis [91]. A study on prenatal PFAS exposure suggested that sphingolipid alterations due to PFASs could lead to type 1 diabetes later in life [92]. Sphingomyelins are another class of sphingolipids that are altered on PFAS exposure and have been associated with insulin resistance, liver dysfunction, and obesity [88]. These findings suggest that PFAS-induced altered sphingolipid metabolism could drive cells towards morphological damage and apoptosis, and can contribute to an insulin-resistant phenotype. Overall, the observed changes in amino acid and lipid metabolism in this study match findings from other PFAS studies [87], suggesting that the metabolic network and modeling approaches applied here can capture PFAS-induced alterations.

**Figure 7:**
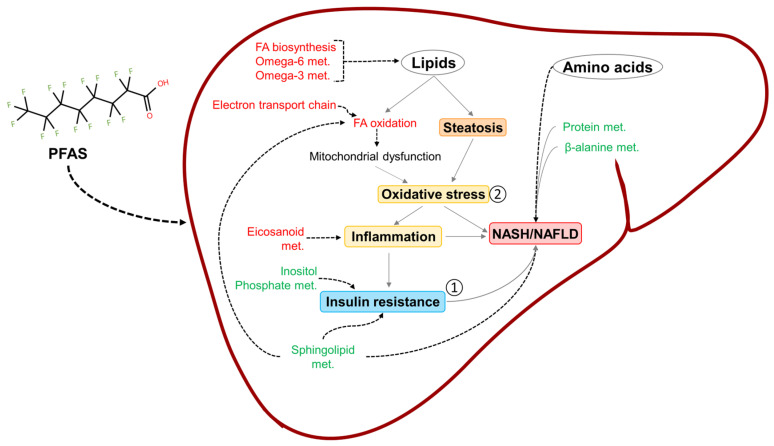
Overview of our findings. PFAS chemicals affect pathways of lipid, energy, and amino acid metabolism that can lead to inflammation, oxidative stress, insulin resistance, and non-alcoholic fatty liver disease (NAFLD). Subsystem names in green and red denote decreased and increased activity, respectively. Dotted lines connect the PFAS-induced changes with existing knowledge of liver diseases. Solid gray lines show disease progression, as reported in the literature. The two hits of NAFLD (insulin resistance and oxidative stress) are marked as 1 and 2. FA: fatty acid; met.: metabolism; NASH: non-alcoholic steatohepatitis; and PFASs: per- and polyfluoroalkyl substances.

Interestingly, our results showed that PFAS exposure decreased inositol phosphate metabolism (Figure 4C), which could contribute to insulin resistance [93], the first hit for NAFLD development. Additional environmental factors, such as diet and medication, could trigger the second hit for NAFLD, which typically involves disruption of fatty acid oxidation and accumulation of reactive oxygen species (ROS) [94,95,96]. The results of this study showed an increase in fatty acid oxidation and the electron transport chain, which can lead to ROS production and subsequently oxidative stress [97,98], the second hit for NAFLD. Notably, the BMD for fatty acid oxidation in males exposed to 6:1 FTOH was smaller than the BMD for inositol phosphate metabolism. Additionally, there were other NAFLD-associated metabolic disruptions that occurred in parallel and with similar dose responses, including an increase in fatty acid biosynthesis, omega-6 fatty acid metabolism, and omega-3 fatty acid metabolism, which disrupt lipid concentrations in the liver and could contribute to steatosis [99]. The increase in eicosanoid metabolism could contribute to inflammation and even hepatocellular carcinoma [100]. Finally, alterations in sphingolipid metabolism have also been associated with insulin resistance and NAFLD [101,102,103,104]. From these observations, we hypothesize that PFAS-induced NAFLD likely follows the “multiple parallel hits theory” rather than the “two-hit theory” [105]. Based on this hypothesis, PFASs would induce multiple mechanisms in parallel, including an imbalance in fatty acids, insulin resistance, mitochondrial dysfunction, and oxidative stress, which can lead to NASH/NAFLD. The alterations in pro-inflammatory eicosanoids and amino acid metabolism would further increase the risk of NAFLD development and progression. Figure 7 summarizes our findings in the context of our hypothesis that PFAS exposure leads to altered metabolism of amino acids and lipids that prime the liver for a fatty and insulin-resistant phenotype.

The PCA plots for the sex-combined analysis of the PFAS-exposed rats showed that the metabolic flux predictions cluster by sex and within each sex cluster by exposure dose. However, our observations of the specific metabolic subsystem alterations showed that PFASs affected similar pathways in both sexes, with differences in relation to the dose of exposure. Male rats showed a higher magnitude of change (higher z-scores) for each subsystem perturbation than female rats across different PFAS chemicals and exposure dose levels, suggesting that the variation between males and females exposed to PFASs is due to the differences in magnitude of change and that dose sensitivity is sex-dependent. Of the PFAS chemicals, 6:1 FTOH induced the largest z-score changes in both sexes (Figure 4 and Figure S3). One mechanism that could account for the observed higher sensitivity in males could be potential differences in PFAS elimination rates between the two sexes. Other studies have reported that females eliminate PFAS compounds much faster than males, leading to lower PFAS bioaccumulation in females [106,107]. In this case, female rats would require higher concentrations of PFASs to elicit a similar response as observed for male rats. Some subsystems, such as porphyrin metabolism, vitamin E metabolism, protein metabolism, and xenobiotics metabolism, responded differently to PFAS types, suggesting that PFAS type also influences the magnitude of alteration. Exposure to 10:2 FTOH caused a large dose-dependent increase in glutathione metabolism and arachidonic acid metabolism that was absent in the other two PFAS compounds, possibly due to carcinogenic mechanisms or to 10:2 FTOH being a long-chain PFAS. Interestingly, 10:2 FTOH can bio-transform into the phased-out PFOA [17], which may indicate that the liver’s metabolic responses to high doses of 10:2 FTOH could resemble those to PFOA. To further compare the PFAS chemicals’ effects between males and females, the differences in the BMDs predicted for each chemical were plotted (Figure 6), which revealed that some PFAS chemicals had a greater sex dependence than others. These results could indicate that the sex-dependent effects are more important for some PFAS types than others. This and our observation that metabolic activity in the liver remained sexually dimorphic even upon exposure to PFAS chemicals (Supplementary Figure S1) reiterate the importance of studying exposures in both sexes, particularly to understand the sex-based outcomes.

Our analysis of control rat gene expression showed that some of the metabolic subsystems showed sex-dependent activity (Figure 2). The female-biased subsystems included central carbon metabolism, carbohydrate metabolism, and the electron transport chain, which influence energy production in the liver. Other studies have also reported that energy metabolism and amino acid usage for energy production are sexually dimorphic [52,108,109]. The discrepancies in the specific amino acid usage between the sexes could be due to differences in experimental conditions, such as animal diet or age. The influence of these factors on sex-dependent liver metabolism requires further experiments. The female-biased subsystems also included bile acid metabolism, which is known to be sex-dependent [110]. The enterohepatic circulation of bile acids has been associated with PFAS accumulation in the liver [111], and it is possible that the increased bile acid metabolism in female rats allows them to clear PFASs faster than male rats. Although the results presented with respect to sexual dimorphism in the control rats are not enough to confirm whether these sex-dependent subsystems protect either sex from liver injury, they do provide hypotheses for experimental validation.

This study has potential limitations with regard to the PFAS exposure setting, rat to human translation, and using transcriptomics to predict the true fluxome. First, we analyzed the effect of a 5-day daily exposure to a single PFAS, which may be different from exposure scenarios for humans, particularly when the latter involves PFAS mixtures and long-term or chronic durations. While the 5-day exposure duration may not fully represent the steady-state or adaptive responses that would occur during chronic, lifetime exposure, it provides a critical snapshot of the initial molecular perturbations in response to the chemicals. It is plausible that some of the observed gene expression changes reflect an initial stress response that could be attenuated over time via homeostatic mechanisms, such as the induction of metabolic enzymes or compensatory feedback loops. Conversely, prolonged exposure could lead to the exhaustion of these adaptive capacities, resulting in different or more severe pathological outcomes not predicted by this short-term profile. Therefore, these findings should be interpreted as a hypothesis-generating exploration of the chemical’s initial mechanism of action rather than a definitive prediction of chronic disease. Furthermore, previous studies have shown that the toxicological potency evaluations from short-term in vivo gavage studies, such as the one described here, are a reasonable approximation, from the standpoint of toxicological potency, of the traditional long-term toxicological assessments [59,60,61,112]. This suggests that the mechanisms predicted here likely describe the early adaptations to PFASs and underlie the initiation of the chemicals’ long-term effects. Second, translating findings from rodents to humans is a challenge that is not unique to this study. Here, we studied the effect of single PFAS exposures on rat livers, while human exposures involve PFAS mixtures as well as possible chronic exposures (compared to the 5-day acute exposure studied here). Translating our findings to humans would require the availability of similar transcriptomic datasets. We could apply our framework, for example, by utilizing liver tissue samples from cadavers, measuring gene expression and PFAS concentrations in the tissue, integrating the gene expression data with a human metabolic model (such as RECON3D [113]), and relating the PFAS concentrations with the predicted metabolic fluxome. Additional information from histopathology of the same tissue would help connect the predicted metabolic state with the histopathological observations. Such experiments would be particularly useful to study the variance within human populations. Alternatively, hepatocyte cell line data could be integrated with hepatocyte/liver-specific metabolic models (such as iHepatocyte [44]), which would be a more controlled experiment and may provide more insights into human-relevant mechanisms. These experiments could also be designed to consider human-relevant doses and chronic exposure conditions, which were not addressed in the current study. Finally, our study did not consider molecular processes, such as gene regulation, translation, posttranslational modifications, and protein degradation. Here, we used transcriptomic data, which may not reflect the true enzyme concentrations in the tissue, limiting the accuracy of the fluxome predictions from Pheflux [58]. While some of our results match proteomics work on PFAS exposures [114], our approach, particularly Pheflux optimization and the consequent BMD predictions, would be more accurate with proteomic measurements.

Future experiments could apply our approach in combination with histopathology to correlate the computational findings with biological observations. Since our approach predicts metabolic activity within the liver, we propose that we validate our findings using metabolomics of liver tissues exposed to these PFASs rather than serum measurements. The levels of ROS, cholesterols, glutathione, branched-chain amino acids, bile acids, and sphingolipids would confirm their roles in PFAS-induced fatty liver and insulin resistance as hypothesized here. Furthermore, validating the findings would inform us of therapeutic strategies to overcome PFAS adverse outcomes in the liver. Based on the BMD calculations, our approach provides the mechanistic insights required for characterizing toxicity and can be applied to rapidly design specific and effective therapeutics. In contrast, traditional apical endpoints, such as organ weight, cell death, and body weight, lack information on the mechanisms underlying the outcomes. In addition to supporting traditional BMDs for regulating safe exposure limits, the BMD values predicted here can serve as a guide to design additional experiments with correct dose spacing for chemical risk assessment and can be correlated with sera chemical concentration measurements to predict the stage of adversity. Finally, the pathways with the lowest BMDs can be used as a guide to identify susceptible individuals in a population. Furthermore, our study did not include the influence of diet and medication, which are known to increase the risk of developing liver injury [115,116]. We hypothesize that the metabolic alterations induced by PFASs increase the burden on male and female livers (Figure 7), consequently increasing the risk of hepatic injury and contributing to NAFLD progression. Further experimentation is needed to confirm the importance of these metabolic subsystems in PFAS-induced hepatic injury and progression.

## 5. Conclusions

Identifying metabolic alterations that precede and occur during toxic responses is important for risk assessment of chemicals and the development of countermeasures. Here, we presented a novel approach for predicting metabolic risk due to chemical exposures and applied it to understand PFAS toxicity mechanisms. We used a gene expression dataset from a 5-day acute exposure study that focused on three PFAS chemicals, including two carboxylic acid-type fluorotelomers and one sulfonamide-type PFAS. The interpretation of metabolic model fluxes and consequently the calculated z-score values requires caution, as some of the parameters, such as those relating to diet, do not exactly match the experimental conditions. While precise constraints would predict more accurate flux values, the overall metabolic predictions may not differ from the current results since the Pheflux algorithm was designed to predict the fluxome closest to the gene expression distribution [58]. The fact that our results agree with existing PFAS exposure studies also corroborates this. Finally, our study is the first to integrate BMD modeling with metabolic fluxes to identify metabolic points of departure. Although the robustness of combining the two methods requires further assessment, it paves the way for computational approaches that can generate more hypotheses and predictions for chemical exposure-induced risk assessment.

## Figures and Tables

**Table 1:** Summary of PFAS chemicals and the doses selected for testing.

PFAS Chemical	CASRN	PubChem CID	OPERA LD_50_ Prediction (Uncertainty Range), mg/kg/Day	U.S. EPA Estimated POD (Uncertainty Range), mg/kg/Day	Selected Dose Levels, mg/kg
6:1 FTOH	375-82-6	550386	460 (230–918)	85 (0.6–637)	0, 0.15, 0.50, 1.40, 4, 12, 37, 111, 333, 1000
10:2 FTOH	865-86-1	70083	636 (319–1270)	18 (0.3–197)	0, 0.07, 0.20, 0.70, 2, 6, 18, 55, 160, 475
PFHxSAm	41997-13-1	11603678	263 (131–525)	35 (0.9–916)	0, 0.15, 0.50, 1.40, 4, 12, 37, 111, 333, 1000

EPA, Environmental Protection Agency; FTOH, fluorotelomer alcohol; LD_50_, median lethal dose; OPERA, OPEn structure–activity/property Relationship App; PFASs, per- and polyfluoroalkyl substances; PFHxSAm, perfluorohexanesulfonamide; and POD, point of departure.

**Table 2:** Benchmark doses for the top PFAS-altered pathways in male and female rats.

Metabolic Subsystem	Male			Female		
	6:1 FTOH	10:2 FTOH	PFHxSAm	6:1 FTOH	10:2 FTOH	PFHxSAm
β-alanine metabolism *	6.6		55.8	260.4	49.0	
Cysteine and methionine metabolism			37.5	33.0	23.4	59.1
Eicosanoid metabolism *	7.7	66.2	54.6	312.9		
Electron transport chain *			3.9	304.6		
Fatty acid biosynthesis *	4.8			12.9	251.7	
Fatty acid metabolism *	4.0			298.8	317.5	63.0
Fatty acid oxidation *	2.3	21.9	20.6	107.8	191.2	31.0
Glutathione metabolism	39.9	10.1		20.0	10.5	
Inositol phosphate metabolism *	4.3					
Nucleotide metabolism	4.0		11.8	76.8		22.3
Omega-3 fatty acid metabolism			22.5	75.5		
Omega-6 fatty acid metabolism	1.6	36.2	18.9			31.0
Porphyrin metabolism		9.6				
Protein metabolism	5.0					
Purine metabolism	2.0	9.8	8.5	36.2	10.0	7.4
Serotonin and melatonin biosynthesis	17.7		22.5		618.9	17.6
Sphingolipid metabolism *	2.9	13.1	35.1		12.2	
Tyrosine metabolism	4.3	70.8		145.4		12.3
Ubiquinone synthesis	3.8	8.4	20.4	23.8		18.4
Valine, leucine, and isoleucine metabolism	1.4			159.5		20.1
Xenobiotic metabolism		16.4	40.1		23.9	

FTOH: fluorotelomer alcohol; PFHxSAm: perfluorohexanesulfonamide. Asterisks (*) mark the common subsystems between the 6:1 FTOH, 10:2 FTOH, and PFHxSAm exposures. If no value is given, then no viable models were found.

## Data Availability

The datasets presented in this study are derived from a previously published study that can be found on the GEO database (accession no. GSE183705). The derived datasets are not readily available but may be obtained following a written request to the corresponding author, along with a summary of the planned research.

## Supplementary Materials

The following supporting information can be downloaded at https://www.mdpi.com/article/10.3390/toxics13080684/s1, Figure S1: Sex-combined principal component analysis of metabolic fluxes in PFAS-exposed rats; Table S1: Ranking of the top features extracted by principal component analysis for each chemical and sex; Figure S2: Z-score changes in the PFAS common subsystems; Figure S3: PFAS-induced change in fluxes of all the metabolic subsystems in iRnov4.2; File S1: Metabolic subsystem annotations in the model; File S2: iRnov4.2 in XML format; File S3: BMD results.

## Abbreviations

The following abbreviations are used in this manuscript:

PFASs	Per- and polyfluoroalkyl substances
GEM	Genome-scale metabolic model
BMD	Benchmark dose
PFCA	Perfluoroalkyl carboxylic acid
PFSA	Perfluoroalkyl sulfonate
PFOA	Perfluorooctanoic acid
PFOS	Perfluorooctanesulfonic acid
NAFLD	Non-alcoholic fatty liver disease
GPR	Gene–protein–reaction
EPA	Environmental Protection Agency
BMDS	Benchmark dose software
NIEHS	National Institute of Environmental Health Sciences
FTOH	Fluorotelomer alcohol
PFHxSAm	Perfluorohexanesulfonamide
PCA	Principal component analysis
BMR	Benchmark response
SD	Standard deviation
PC	Principal component
NASH	Non-alcoholic steatohepatitis
ROS	Reactive oxygen species
