# Supplementary material for: Genome-Scale Metabolic Modeling Predicts Per- and Polyfluoroalkyl Substance-Mediated Early Perturbations in Liver Metabolism
Source: Toxics. 2025 Aug 17;13(8):684. doi: 10.3390/toxics13080684 (PMC12390441; doi:10.3390/toxics13080684)
Supplement: Supplementary file 1 [file toxics-13-00684-s001.zip › Supplementary material.pdf]

Supplementary material

Genome-scale metabolic modeling predicts PFAS-mediated early perturbations in liver metabolism

Archana Hari[1,2], Michele R. Balik-Meisner[3], Deepak Mav[3], Dhiral P. Phadke[3], Elizabeth H. Scholl[3], Ruchir R. Shah[3], Warren Casey[4], Scott S. Auerbach[4], Anders Wallqvist[1,*], and Venkat R. Pannala[1,2*]

[1]Department of Defense Biotechnology High Performance Computing Software Applications Institute, Defense Health Agency Research & Development, Medical Research and Development Command, Fort Detrick, MD, USA
[2]The Henry M. Jackson Foundation for the Advancement of Military Medicine, Inc., Bethesda, MD, USA
[3]Sciome LLC, Research Triangle Park, NC, USA
[4]Division of Translational Toxicology, National Institute of Environmental Health Sciences, Research Triangle Park, NC, USA

*Correspondence: sven.a.wallqvist.civ@health.mil; Tel.: (301) 619-1989 (A.W.); vpannala@bhsai.org; Tel.: (301) 619-1978 (V.R.P.)

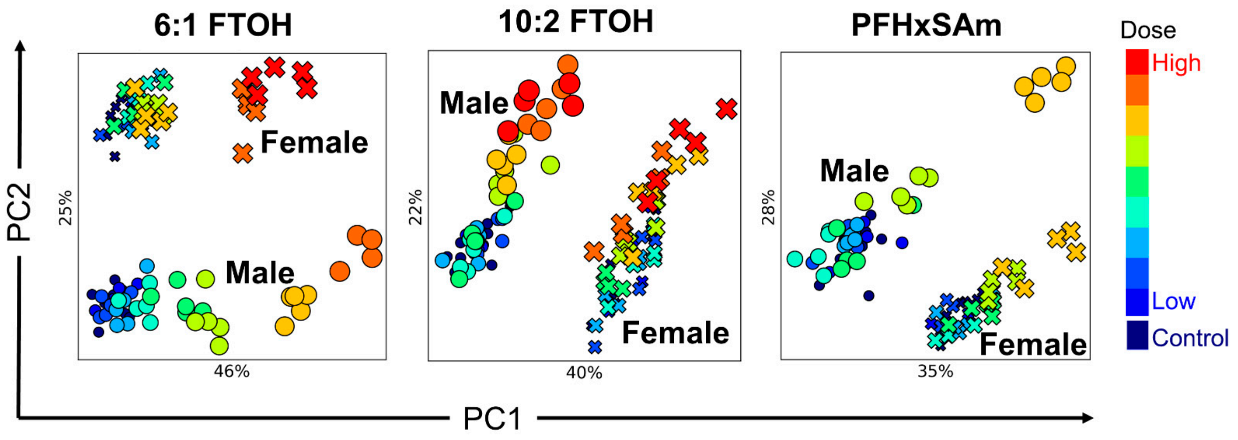

Supplementary Figure S1. Sex-combined principal component analysis of metabolic fluxes in PFAS-exposed rats. Circles represent male rats, and crosses represent female rats. Colors indicate exposure doses. FTOH: fluorotelomer alcohol; PFHxSAm: perfluorohexanesulfonamide; PC: principal component.

Supplementary Table S1. Ranking of the top features extracted by principal component analysis for each chemical and sex.

Metabolic subsystem	Male			Female		
	6:1 FTOH	10:2 FTOH	PFHxSAm	6:1 FTOH	10:2 FTOH	PFHxSAm
β-Alanine metabolism	4	-	8	-	9	-
Cysteine and methionine metabolism	-	-	-	10	-	-
Fatty acid biosynthesis	2	2	6	2	3	5
Fatty acid metabolism	10	-	-	5	10	
Fatty acid oxidation	1	1	1	1	4	1
Glutathione metabolism	-	3	-	-	-	-
Inositol phosphate metabolism	9	6	-	6	2	2
Nucleotide metabolism	-	-	-	9	-	-
Omega-3 fatty acid metabolism	-	4	3	-	-	-
Omega-6 fatty acid metabolism	-	7	4	-	-	-
Porphyrin metabolism	-	10	-	-	-	-
Protein metabolism	-	-	-	8	6	4
Purine metabolism	5	-	2	-	-	10
Serotonin and melatonin biosynthesis	-	-	-	-	-	8
Sphingolipid metabolism	3	8	9	7	1	-
Tyrosine metabolism	-	-	-	-	-	9
Ubiquinone synthesis	-	-	-	-	-	6
Valine, leucine, and isoleucine metabolism	6	-	10	4	-	3
Vitamin E metabolism	-	-	-	-	8	-
Xenobiotic metabolism	-	-	-	-	5	-

FTOH: fluorotelomer alcohol; PFHxSAm: perfluorohexanesulfonamide.

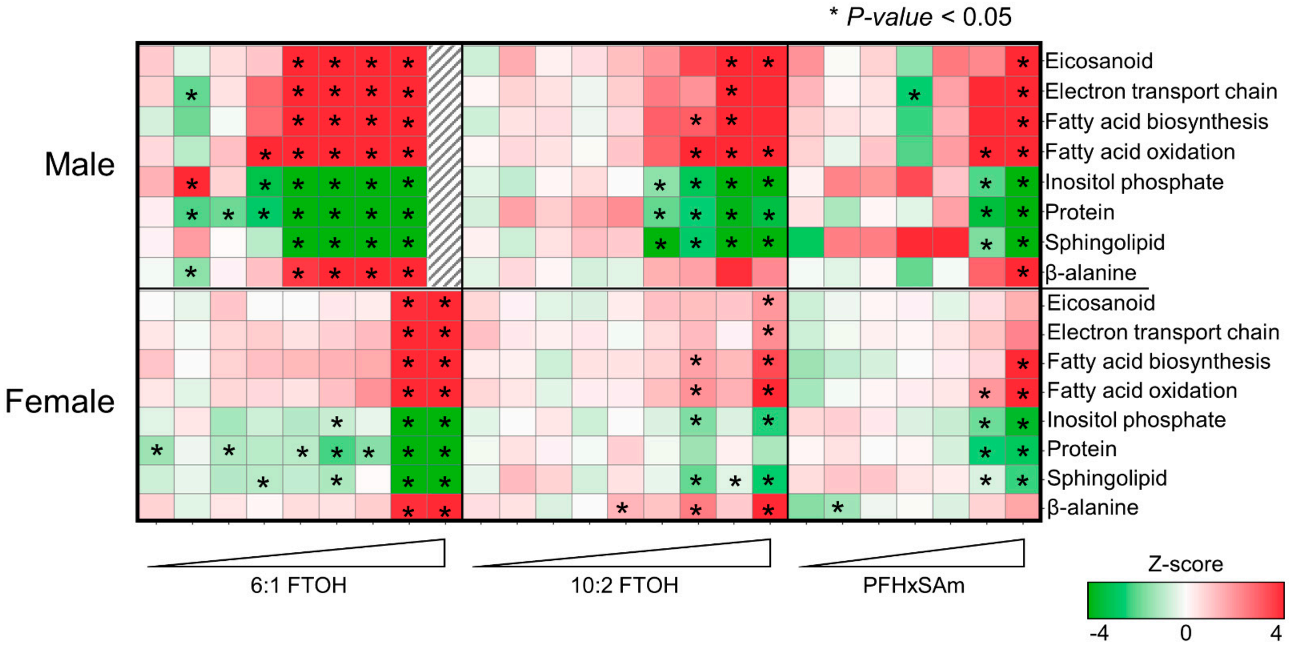

Supplementary Figure S2. Z-score changes in the PFAS-common subsystems. Asterisks (*) mark the changes that are statistically significant (P-value < 0.05) by the Mann-Whitney U test. Triangles depict increasing PFAS doses. Metabolic subsystem labels are on the y-axis. FTOH: fluorotelomer alcohol; PFHxSAm: perfluorohexanesulfonamide.

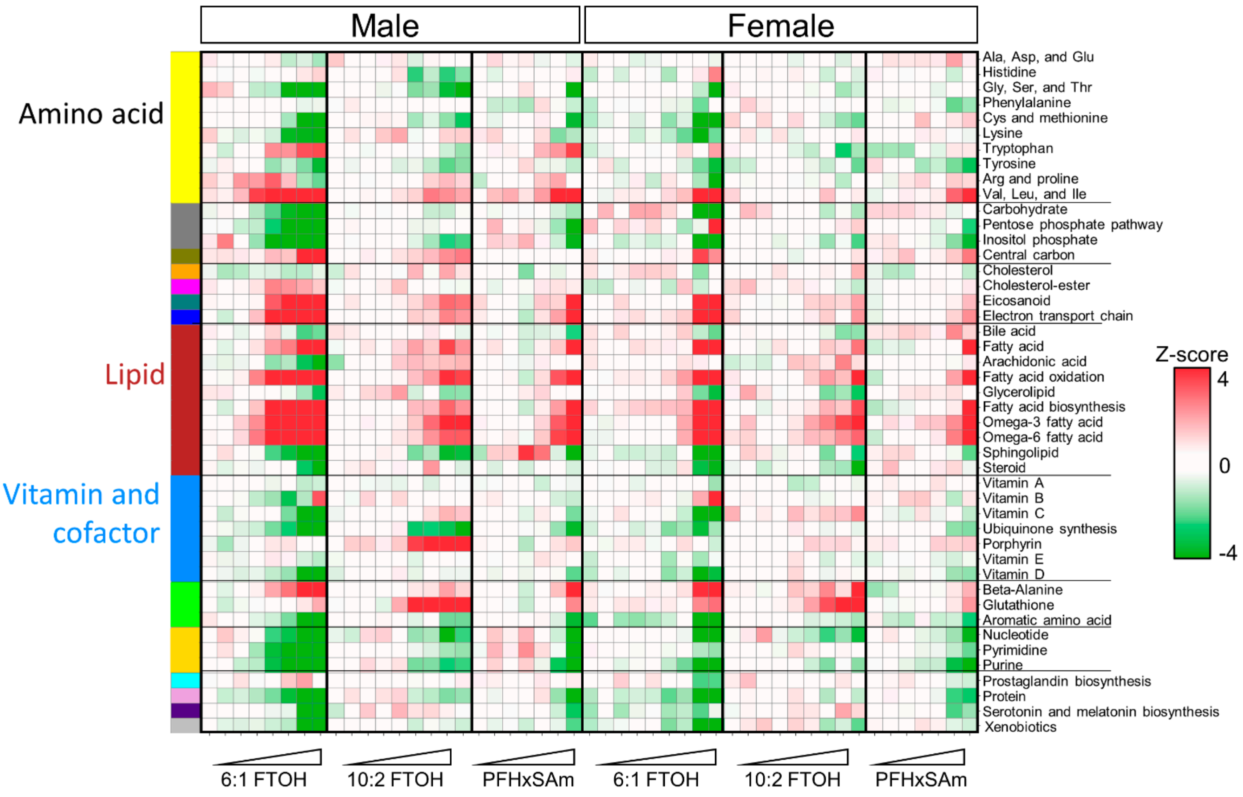

Supplementary Figure S3. PFAS-induced change in fluxes of all the metabolic subsystems in *iRnov4.2*. *Z*-scores for each subsystem are with respect to fluxes in control rats. Triangles depict increasing PFAS doses. Colored ribbon on the left of the heatmap denotes the super-pathway classifications of the subsystems: yellow, amino acid metabolism; gray, carbohydrate metabolism; olive, central carbon metabolism; brown, lipid metabolism; sky blue, vitamin and cofactor metabolism; sage green, metabolism of other amino acids; gold, nucleotide metabolism. Other pathways contain individual subsystems labeled on the y-axis: Ala, alanine; Asp, aspartate; Glu, glutamate; Gly, glycine; Ser, serine; Thr, threonine; Cys, cysteine; Arg, arginine; Val, valine; Leu, leucine; Ile, isoleucine. FTOH: fluorotelomer alcohol; PFHxSAm: perfluorohexanesulfonamide.
